# Post-mortem histology in transient receptor potential cation channel subfamily V member 6 (TRPV6) under-mineralising skeletal dysplasia suggests postnatal skeletal recovery: a case report

**DOI:** 10.1186/s12881-020-01007-z

**Published:** 2020-03-30

**Authors:** Anna E. Mason, David Grier, Sarah F. Smithson, Christine P. Burren, Elise Gradhand

**Affiliations:** 1 Bristol Medical School Translational Health Sciences, University of Bristol, Bristol, UK; 2 Department of Radiology, Bristol Royal Hospital for Children, University Hospitals Bristol NHS Foundation Trust, Bristol, UK; 3 Department of Clinical Genetics, St Michaels Hospital, University Hospitals Bristol NHS Foundation Trust, Bristol, UK; 4 Department of Paediatric Endocrinology, Bristol Royal Hospital for Children, University Hospitals Bristol NHS Foundation Trust, Bristol, UK; 5 Severn Pathology, Paediatric and Perinatal Pathology, Southmead Hospital, North Bristol NHS Trust, Bristol, UK; 6 Dr. Senckenberg. Institut für Pathologie, Universitätsklinikum Frankfurt/Main, Theodor-Stern-Kai 7, 60590 Frankfurt, Germany

**Keywords:** TRPV6 (transient receptor potential cation channel subfamily V member 6), Skeletal dysplasia, Placental calcium transfer, Post-mortem

## Abstract

**Background:**

The calcium-selective channel TRPV6 (transient receptor potential cation channel subfamily V member 6) is crucial for maternal-fetal calcium transport across the placenta. *TRPV6* mutations have recently been associated with an antenatally severe under-mineralising skeletal dysplasia accompanied by postnatal biochemical abnormalities. This is the first post-mortem report in a patient with *TRPV6* skeletal dysplasia.

**Case presentation:**

The female infant had severe antenatal and postnatal skeletal abnormalities by 20 weeks gestation and was ventilator-dependent from birth. These skeletal abnormalities were apparent at an earlier gestational age than in previous reported cases and a more severe clinical course ensued. Biochemical and skeletal abnormalities, including bone density, improved postnatally but cardiac arrest at 4 months of age led to withdrawal of intensive care. Compound heterozygous *TRPV6* variants (c.1978G > C p.(Gly660Arg) and c.1528C > T p.(Arg510Ter)) were identified on exome sequencing. Post-mortem identified skeletal abnormalities but no specific abnormalities in other organ systems. No placental pathology was found, multi-organ histological features reflected prolonged intensive care only. Post-mortem macroscopic examination indicated reduced thoracic size and short, pale and pliable ribs. Histological examination identified reduced number of trabeculae in the diaphyses (away from the growth plates), whereas metaphyses showed adequate mineralisation and normal number of trabeculae, but with slightly enlarged reactive chondrocytes, indicating post-natal skeletal growth recovery. Post-mortem radiological findings demonstrated improved bone density, improved rib width, healed fractures, although ribs were still shorter than normal. Long bones (especially humerus and femur) had improved from initial poorly defined metaphyses and reduced bone density to sharply defined metaphyses, prominent growth restart lines in distal diaphyses and bone-in-bone appearance along diaphyses.

**Conclusions:**

This case provide bone histological confirmation that human skeletal development is compromised in the presence of *TRPV6* pathogenic variants. Post-mortem findings were consistent with abnormal in utero skeletal mineralisation due to severe calcium deficit from compromised placental calcium transfer, followed by subsequent phenotypic improvement with adequate postnatal calcium availability. Significant skeletal recovery occurs in the early weeks of postnatal life in TRPV6 skeletal dysplasia.

## Background

Calcium is essential for cell signalling, neuromuscular activity, blood coagulation and skeletal growth. During fetal development, additional calcium is required to support skeletal formation and development. This demand is met by active maternal-foetal (transplacental) calcium transport, to achieve higher fetal than maternal serum calcium concentration [1]. Various mechanisms have been proposed, with recent recognition of the importance of transient receptor potential cation channel subfamily V member 6 (TRPV6; also referred to as vanilloid), an epithelial calcium-selective channel formed of four identical subunits, each with six transmembrane segments [2]. *TRPV6* gene location and expression varies across species. In humans, the *TRPV6* gene is located on chromosome 7q33-q34 and is very highly expressed in the placenta [3].

Alteration in *TRPV6* expression contributes to many pathological processes, but only recently has a human phenotype been identified. *TRPV6* is upregulated in several human malignancies, including prostate, thyroid, breast and endometrial cancer, where it aids the calcium-dependent proliferation of malignant cells [4–6]. Conversely, it is downregulated in pre-eclampsia, a condition associated with reduced placental calcium transfer [7]. A contributory potential role for *TRPV6* has also been postulated in several rare childhood disorders including Lowe, Pendred, Gitelman and Gordon syndromes; although those disorders involve additional electrolyte abnormalities, not present in this case, suggesting multiple mechanisms within those disorders [8–11]. Prior to 2018, *TRPV6* had not been identified in human skeletal disease.

Very recently in 2018, homozygous or compound heterozygous *TRPV6* variants have been reported in seven infants with a skeletal disorder of antenatal-onset characterised by poor mineralisation [12, 13]. All seven infants had biochemical abnormalities of secondary neonatal hyperparathyroidism which completely normalised over several weeks. The skeletal abnormalities improved to varying extents over a longer timeframe during infancy. Our patient [12] showed more severe skeletal abnormalities than the other six cases, and much earlier in pregnancy. The variants in our case were different and the greater clinical severity is interpreted to have been the consequence of the different location of the pathogenic variants in this case compared to the other 6 causing more severe impact on TRPV6 protein function. Ultimately, this infant did not survive, due to the initial postnatal respiratory impairment, ineffective respiratory weaning and therefore complications of prolonged intensive care treatment. The detailed genetic findings on this infant were published in the American Journal of Medical Genetics 2018 and we refer the reader back to that publication for detail on in silico modelling and rationale of pathogenicity according to ACMG Guidelines. Whereas, in this publication, we focus on the post-mortem findings of this case, which are the first in *TRPV6*-associated skeletal dysplasia, shedding new insight into the abnormal bone architecture of this condition and skeletal development in the perinatal period.

## Case presentation

A British White female infant had severe, potentially lethal, skeletal dysplasia detected antenatally at 20 weeks’ gestation. This consisted of short long bones, a small chest, and rib deformities suggesting pulmonary hypoplasia. Antenatal microarray comparative genomic hybridisation (CGH) and uniparental disomy (UPD) testing, to exclude paternal UPD14, did not identify abnormalities. She was born at term (39 + 1/40) with normal birth weight (3128 g) and was ventilator-dependent from birth due to respiratory distress. Physical examination showed a bell-shaped chest, but she was otherwise well-grown and had no dysmorphic features or abnormal neurology.

**Fig. 1:**
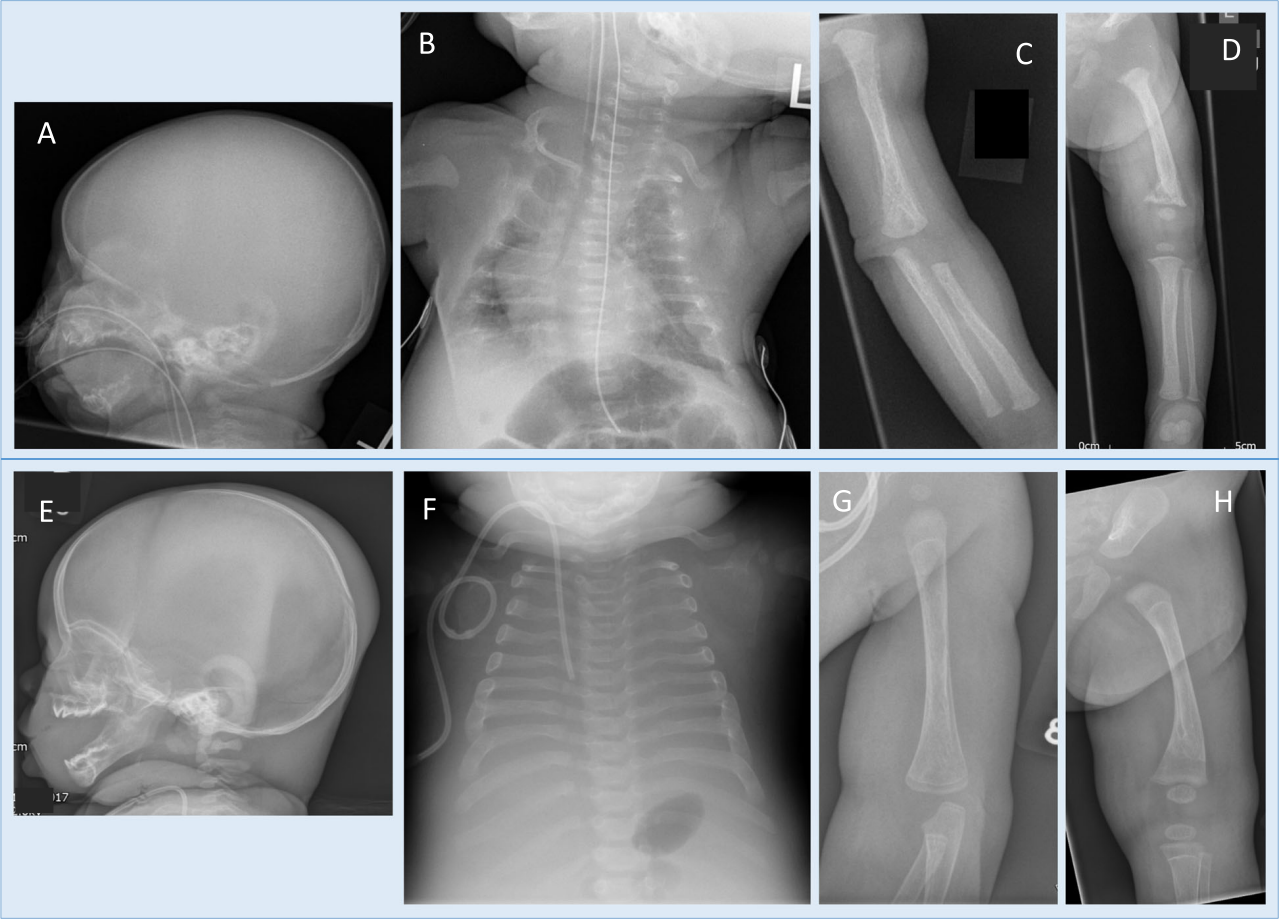
Radiological findings. Upper panel (**a**-**d**) shows early postnatal plain radiographs (Day 12) and lower panel (**e**-**h**) shows corresponding post-mortem plain radiographs. Bone density is considerably less in each upper panel image compared with lower images (skull **a** and **e**, chest **b** and **f**, upper limb **c** and **g**, lower limb **d** and **h**). Chest X-rays (**b** and **f**): thoracic size is extremely reduced (**b**), to a lesser degree post-mortem (**f**), which also shows broad and more calcified ribs, although with irregularities posteriorly and laterally consistent with healed fractures. Upper limb: early postnatal imaging (**c**) shows ill-defined metaphyses; then (**g**) more sharply defined metaphyses, prominent growth restart lines in diaphyses and bone-in-bone appearance. Lower limb: **d** shows initial poorly defined metaphyses, reduced bone density and metaphyseal fractures; then H shows healed fractures, better metaphyseal delineation, prominent growth restart lines in diaphyses and bone-in-bone appearance

Skeletal survey showed persistent generalised under-mineralisation, multiple rib and metaphyseal fractures and periosteal reaction along long bone diaphyses (Fig. 1). This was associated with significantly elevated parathyroid hormone (PTH) during the first 6 weeks (peak 101 pmol/L, reference range 1.1–6.9 pmol/L) but normal corrected calcium, phosphate and alkaline phosphatase (ALP). Postnatal genetic testing, using targeted single gene approach, excluded potential differential diagnoses of Neonatal Severe Hyperparathyroidism, Mucolipidosis Type II (I-cell disease), which can also feature hyperparathyroidism and periosteal changes, and 336 known skeletal dysplasias. Further details can be found in the initial case report publication [12].

Whole exome sequencing was performed with DNA samples from the patient and her unaffected parents using the Agilent SureSelect All Exon v6 system, with sequencing on an Illumina NextSeq 500. These aforementioned negative genetic test finding reports coincided with identifying resolution of PTH elevation and progressively improving skeletal mineralisation. Consequently, we considered an alternative hypothesis of an in utero calcium deficiency caused by an intrinsic defect in placental calcium transfer. This prompted direct exploration of the candidate genes *TRPV6, CABP9K* and *VDR.* We performed a gene-agnostic trio analysis to identify very rare variants (Minor Allele Frequency < 0.0001) compatible with autosomal recessive or de novo inheritance. The likely causative variants were confirmed by Sanger sequencing. 12 This analysis identified compound heterozygous *TRPV6* variants: novel maternally inherited missense variant, c.1978G > C p.(Gly660Arg), and paternally inherited nonsense variant, c.1528C > T p.(Arg510Ter); both classified as pathogenic according to ACMG Guidelines.

By 6 weeks of age, bone mineralisation showed radiological improvement, but ventilatory requirement persisted and was provided via tracheostomy from 8 weeks of age. At 17 weeks, she developed rapid onset marked abdominal distension, followed by two cardiac arrests associated with prolonged lactic acidosis. Prior to this, the cardiac monitoring had shown no evidence of cardiac malfunction or arrhythmias. Emergency laparotomy confirmed the presence of volvulus and dusky, but not ischaemic, bowel. Multi-organ failure, including global ischaemic brain injury, was present and ultimately care was withdrawn.

Post-mortem skeletal survey radiographs were undertaken (Fig. 1). They demonstrated considerably improved bone density compared to early postnatal imaging, metaphyseal regions had been ill-defined on early postnatal imaging, but were far more sharply defined, prominent growth restart lines were present in long bone diaphyses and ossification was in keeping with chronological age. The ribs remained extremely short, were irregular posteriorly and laterally suggesting healed fractures, and the thoracic volume was markedly reduced.

A limited post-mortem autopsy was performed (chest, abdomen, ribs and long bones only). The infant was well-grown: body weight 6370 g, crown-heel length 62 cm, crown-rump length 45 cm and head circumference 43 cm (all within normal range for 4 months of age) with no dysmorphic features, no abnormalities of skin or hair. A narrow chest was noted.

**Fig. 2:**
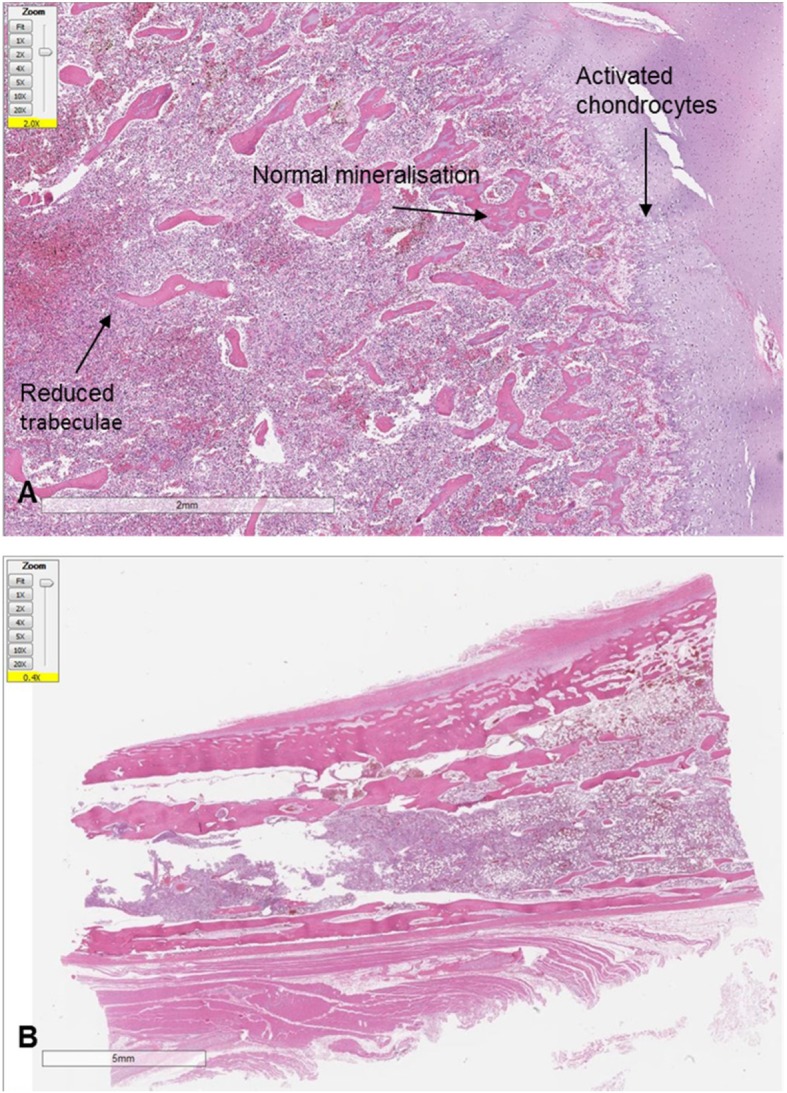
Histological Findings. **a** Histology of the femur at 2x magnification using Haematoxylin and Eosin (H + E) staining. This shows activated and slightly irregular columns of chondrocytes*,* adequately mineralised trabeculae in the metaphyseal region of the endochondral ossification zone*,* but reduced number of trabeculae in the diaphysis (see arrows). **b** Histology of the rib at 2x magnification using H + E staining showing reduction in bony trabeculae of the rib

There was evidence of abnormal rib and femur bone architecture on macroscopic and microscopic examination. The bones were pale, unusually pliable and easily cut away from the costo-cartilaginous junction with a scalpel. Microscopically, the rib and femur metaphyses showed growth plates with the expected three zones (proliferative, hypertrophic and calcified cartilage), but the chondrocytes were relatively enlarged and activated in keeping with increased chondroid matrix production. Towards the diaphyses, the trabeculae were of normal structure but significantly reduced in number (Fig. 2). The bone marrow was mildly autolysed but showed normal cellularity and a normal trilineage haematopoiesis.

Borderline lung hypoplasia was present (lung to body weight ratio 0.017, abnormal < 0.015). Pulmonary microscopic findings were in keeping with multi-organ failure, with abundant alveolar macrophages, parenchymal and intra-alveolar haemorrhage. No histological features of infantile respiratory distress syndrome were seen. On macroscopic examination, the heart was mildly enlarged (weight 30.9 g, expected 23 g for crown-rump length), but structurally normal with no pericardial effusion. Microscopic examination showed mild hypertrophy and disarray of the cardiomyocytes with a normal conduction system. No significant areas of myocardial infarction were noted.

Abdominal examination showed normal macroscopic and microscopic appearance of the intestines with no full thickness necrosis of the bowel or evidence of malrotation. The caecum was mobile and there was a large haemorrhage in the mesentery of the sigmoid colon with approximately 20 ml of free blood in the abdominal cavity in keeping with surgical intervention during emergency laparotomy. The liver showed patchy hepatocyte necrosis and prominent bilirubinostasis. The pancreas appeared macroscopically normal and showed no significant histological abnormality of endocrine and exocrine glands. Immunohistochemical studies were not possible due to the extent of autolysis.

Multisystem histological features consistent with intensive care treatment where present but the exact aetiology leading to cardiac arrest and subsequent multi-organ failure could not be established.

There was no significant placental abnormality (trimmed weight 400 g, 10-25th percentile for gestation), including no focal changes on macroscopic or microscopic examination on routine staining. There was no evidence of viral infection. Immunohistochemical staining for TRPV6 was attempted using three antibodies (AB20C6(C-term), AB429(C-term) and AB1271(N-term)) on formalin-fixed, paraffin-embedded placental tissue. Unfortunately, reliable staining could not be achieved. The antibodies were developed for use on fresh frozen tissue and it appears likely that the placental TRPV6 antigen did not survive formalin fixation, although this has not been formally tested.

In summary, post-mortem pathological examination identified ongoing skeletal abnormality with pale, pliable and short ribs. Post-mortem radiology illustrated considerable improvement in bone density and metaphyseal growth, supported by histological evidence of growth recovery with reactive chondrocytes and adequate mineralised normal trabeculae in the metaphyses, yet trabeculae were sparse but normally structured in the diaphyses.

## Discussion and conclusions

We report the findings at post-mortem of an infant with antenatal-onset skeletal dysplasia caused by compound heterozygous *TRPV6* variants. TRPV6, a calcium-selective channel, plays an important role in maternal-foetal calcium transport via the placenta. Variants in *TRPV6* may lead to insufficient calcium transport and abnormal skeletal development in utero. This leads to postnatal skeletal and biochemical abnormalities which progressively improve in the *ex utero* environment, as described in seven recent cases*.*^12,13^ Skeletal findings improved postnatally in all cases, although in our case [12] the phenotype was extremely severe. In particular, the abnormally small rib cage led to life-threatening respiratory insufficiency. Her ventilator-dependency had been forecast to continue until approximately 18 months of age (her unexpected death aged 4 months meant this duration was ultimately not known). A further difference in this case, is that the skeletal dysplasia was evident much earlier antenatally, whereas in the Suzuki cases skeletal abnormalities were only evident from the third trimester onwards (earliest 28 weeks). Although maternal-foetal calcium transfer begins at 12 weeks gestation, the majority occurs during the third trimester so it had been hypothesised that foetal bone abnormalities would not be evident earlier [14]. We suspect that the significant skeletal dysplasia evident by only 20 weeks’ gestation in this case reflects the severity of the compromise to calcium transfer resulting from severely impaired TRPV6 function. In support of this, the variants in *TRPV6* identified in our patient were predicted, by in silico protein modelling, to result in unstable or misfolded protein which might lead to channel loss or impaired activity.

The post-mortem findings further support the concept of significant under-mineralisation due to lack of calcium, especially the paleness, soft texture and flexibility of the bones. Microscopically, the reduced bony trabeculae persisting in the diaphyses may reflect earlier absent or reduced calcium availability in utero, whereas the reactive chondrocytes in the growth plate suggest bone recovery and catch up in the normal calcium environment after birth. Significant bone remodelling occurs during growth, so the reduction in trabecular number might progressively normalise during childhood. Optimism arises from other recent cases suggesting normal postnatal growth, although duration is currently short with minimal linear growth details. Yet recent *Trpv6* mouse model studies by Fecher-Trost give caution as cortical bone architecture remained abnormal in thickness with reduced femoral length [15].

We consider it likely that the small lung volume was a consequence of the skeletal dysplasia rather than due to a primary pulmonary hypoplasia. This is supported by the short ribs and markedly reduced thoracic volume. Moreover, the lungs showed a normal pattern of lobar development and furthermore there was no histological remodelling of the lung tissue due to the inflammatory changes typically seen in infantile respiratory distress syndrome and, despite evidence of multi-organ failure, there were no other significant pulmonary findings.

All organs were exposed to reduced calcium levels during development, which could conceivably disrupt physiological processes other than skeletal development. The cardiac histology was examined in detail, as calcium is essential for the conduction system and contractility of the cardiomyocytes. The post-mortem findings of a normal conduction system, along with the absence of identified arrhythmias during her first 4 months of life, provide no indication of a primary cardiac abnormality. The mild cardiac hypertrophy and disarray of the cardiomyocytes are non-specific findings and are post-mortem findings not uncommonly seen in post-resuscitation, consistent with recurrent adrenaline and/or corticosteroid administration. This correlates with the intensive care treatment provided in this case rather than a result of an abnormal calcium metabolism. Cardiac arrythmias were not mentioned in the other cases. Our histological findings do not raise abnormalities suggesting the need for cardiac surveillance in surviving infants and children.

Clinically, there was no evidence of wider disorders of calcium homeostasis. There was no pre-eclampsia and no maternal factors that could account for in utero calcium deficiency. Postnatally, oral calcium supplementation at physiological doses was given briefly to compensate for an expected persistent hypocalcaemia due to the skeletal calcium deficiency. However, normocalcaemia was maintained by a normal milk diet only, indicating normal intestinal calcium absorption in the postnatal period. The exocrine pancreas is another key site for *TRPV6* expression in humans, although significance of its role is unclear. Unfortunately, histology in this case was non-contributory due to autolysis. But antemortem, there had been no clinical evidence of pancreatic exocrine gland dysfunction, suggesting its role may be minimal.

As more roles for *TRPV6* in human disease are identified, further studies are vital to improve our understanding of TRPV6 function and identify potential pathways to prevent, modify or treat these disorders. Its upregulation in many malignancies already makes it a promising target for cancer therapy. Several groups have now developed systemic *Trpv6* knock-out mice. The phenotypes are variable between different mouse lines but include male hypofertility, elevated PTH, growth retardation, poor bone mineralisation and dermatitis [16–18]. However, clinical correlation to human disease is not straight forward as organ-specific expression of TRPV6 varies between species. Studying the foetal development of *Trpv6* knock-out mice will be extremely important in advancing our understanding of antenatal calcium homeostasis and identifying potential therapeutic intervention in disorders such as pre-eclampsia and *TRPV6* under-mineralising skeletal dysplasia. While intra-amniotic calcium infusions could theoretically prevent or correct bone abnormalities, improving thoracic growth and optimising future lung function, their risk profile of pregnancy loss means they are not a realistic approach. It has been proposed that small molecular chaperones, such as those used in cystic fibrosis, could be used antenatally to correct the conformation of *TRPV6* variants, although this remains hypothetical at present [13].

In summary, we report the first post-mortem histological and radiological findings of a patient with *TRPV6*-associated skeletal dysplasia. The histology suggests that metaphyses had been compromised due to severe lack of calcium substrate, but then functioned normally, in fact possibly more active, in the postnatal environment. Bone development through collagen formation is intrinsically abnormal in the majority of skeletal dysplasias. *TRPV6*-associated skeletal dysplasia differs where the main feature appears to be compromised development of bone quantity resulting from extreme calcium lack during crucial in utero development. The post-mortem radiology and histology in our case suggested postnatal catch-up and recovery. Although, an effect might persist as the trabeculae remained reduced in number in the diaphyses. Whether bone remodelling throughout longer childhood growth can completely normalise bone length and density is currently unknown and will require close monitoring in other cases.

## Data Availability

DNA sequences are available in ClinVar. Accession number SCV001167185: https://www.ncbi.nlm.nih.gov/clinvar/variation/818220/

## Abbreviations

ALP: Alkaline phosphatase
PTH: Parathyroid hormone
TRPV6: Transient receptor potential cation channel subfamily V member 6
UPD: Uniparental disomy

## References

[1] Kovacs CS, Kronenberg HM (1997). Maternal-fetal calcium and bone metabolism during pregnancy, puerperium, and lactation. Endocr Rev.

[2] Fecher-Trost C, Wissenbach U, Weissgerber P (2017). TRPV6: from identification to function. Cell Calcium.

[3] Moreau R, Daoud G, Bernatchez R, Simoneau L, Masse A, Lafond J (2002). Calcium uptake and calcium transporter expression by trophoblast cells from human term placenta. Biochim Biophys Acta.

[4] Fixemer T, Wissenbach U, Flockerzi V, Bonkhoff H (2003). Expression of the Ca2+−selective cation channel TRPV6 in human prostate cancer: a novel prognostic marker for tumor progression. Oncogene..

[5] Bolanz KA, Hediger MA, Landowski CP (2008). The role of TRPV6 in breast carcinogenesis. Mol Cancer Ther.

[6] Zhuang L, Peng JB, Tou L, Takanaga H, Adam RM, Hediger MA (2002). Calcium-selective ion channel, CaT1, is apically localized in gastrointestinal tract epithelia and is aberrantly expressed in human malignancies. Lab Investig.

[7] Haché S, Takser L, LeBellego F, Weiler H, Leduc L, Forest JC (2011). Alteration of calcium homeostasis in primary preeclamptic syncytiotrophoblasts: effect on calcium exchange in placenta. J Cell Mol Med.

[8] Yang SS, Lo YF, Yu IS, Lin SW, Chang TH, Hsu YJ (2010). Generation and analysis of the thiazide-sensitive Na+ −cl- cotransporter (Ncc/Slc12a3) Ser707X knockin mouse as a model of Gitelman syndrome. Hum Mutat.

[9] Yang SS, Hsu YJ, Chiga M, Rai T, Sasaki S, Uchida S (2010). Mechanisms for hypercalciuria in pseudohypoaldosteronism type II-causing WNK4 knock-in mice. Endocrinology..

[10] Nakaya K, Harbidge DG, Wangemann P, Schultz BD, Green ED, Wall SM (2007). Lack of pendrin HCO3- transport elevates vestibular endolymphatic [Ca2+] by inhibition of acid-sensitive TRPV5 and TRPV6 channels. Am J Physiol Renal Physiol.

[11] Wu G, Zhang W, Na T, Jing H, Wu H, Peng JB (2012). Suppression of intestinal calcium entry channel TRPV6 by OCRL, a lipid phosphatase associated with Lowe syndrome and dent disease. Am J Physiol Cell Physiol.

[12] Burren CP, Caswell R, Castle B, Welch CR, Hilliard TN, Smithson SF (2018). TRPV6 compound heterozygous variants result in impaired placental calcium transport and severe undermineralization and dysplasia of the fetal skeleton. Am J Med Genet Part A.

[13] Suzuki Y, Chitayat D, Sawada H, Deardorff MA, McLaughlin HM, Begtrup A (2018). TRPV6 variants interfere with maternal-fetal calcium transport through the placenta and cause transient neonatal hyperparathyroidism. Am J Hum Genet.

[14] Hacker AN, Fung EB, King JC (2012). Role of calcium during pregnancy: maternal and fetal needs. Nutr Rev.

[15] Fecher-Trost C, Lux F, Busch K, Raza A, Winter M, Hielscher F (2019). *Maternal* transient receptor potential Vanilloid 6 (Trpv6) is involved in offspring bone development. J Bone Miner Res.

[16] Chen F, Ni B, Yang YO, Ye T, Chen A (2014). Knockout of TRPV6 causes osteopenia in mice by increasing osteoclastic differentiation and activity. Cell Physiol Biochem.

[17] Weissgerber P, Kriebs U, Tsvilovskyy J, Olausson J, Kretz O, Stoerger C, Mannebach S (2012). Excision of the *Trpv6* gene leads to severe defects in epididymal Ca2+ absorption and male infertility much alike the single D541A pore mutation. J Biol Chem.

[18] Bianco SD, Peng JB, Takanaga H, Suzuki Y, Crescenzi A, Kos CH (2007). Marked disturbance of calcium homeostasis in mice with targeted disruption of the *Trpv6* calcium channel gene. J Bone Miner Res.

